# Skeletal Correlates for Body Mass Estimation in Modern and Fossil Flying Birds

**DOI:** 10.1371/journal.pone.0082000

**Published:** 2013-11-29

**Authors:** Daniel J. Field, Colton Lynner, Christian Brown, Simon A. F. Darroch

**Affiliations:** Department of Geology and Geophysics, Yale University, New Haven, Connecticut, United States of America; University of Lethbridge, Canada

## Abstract

Scaling relationships between skeletal dimensions and body mass in extant birds are often used to estimate body mass in fossil crown-group birds, as well as in stem-group avialans. However, useful statistical measurements for constraining the precision and accuracy of fossil mass estimates are rarely provided, which prevents the quantification of robust upper and lower bound body mass estimates for fossils. Here, we generate thirteen body mass correlations and associated measures of statistical robustness using a sample of 863 extant flying birds. By providing robust body mass regressions with upper- and lower-bound prediction intervals for individual skeletal elements, we address the longstanding problem of body mass estimation for highly fragmentary fossil birds. We demonstrate that the most precise proxy for estimating body mass in the overall dataset, measured both as coefficient determination of ordinary least squares regression and percent prediction error, is the maximum diameter of the coracoid’s humeral articulation facet (the glenoid). We further demonstrate that this result is consistent among the majority of investigated avian orders (10 out of 18). As a result, we suggest that, in the majority of cases, this proxy may provide the most accurate estimates of body mass for volant fossil birds. Additionally, by presenting statistical measurements of body mass prediction error for thirteen different body mass regressions, this study provides a much-needed quantitative framework for the accurate estimation of body mass and associated ecological correlates in fossil birds. The application of these regressions will enhance the precision and robustness of many mass-based inferences in future paleornithological studies.

## Introduction

In vertebrates, body mass is known to influence many important biological parameters, ranging from physiological [1,2], to biomechanical [3-6], to ecological [7,8]. As such, accurate predictions of body mass are crucial to understanding how ancient organisms lived, and for understanding how body mass and its attendant biological correlates have evolved over time. Accurate, repeatable estimators of body mass for fossil taxa are particularly important to the study of bird evolution, given the large number of recently discovered crownward stem birds--these Mesozoic fossils have greatly informed our understanding of the character transitions within Maniraptora that ultimately gave rise to extant birds [9]. Examples of body mass-dependent character transitions on the bird stem include the evolution of increased avian encephalization quotients [10-12], homeothermy [13], body size [14-16], and flight [17-19].

Given the broad paleobiological significance of estimating body mass from extant bird skeletons, several studies have presented scaling relationships between avian body mass and various skeletal dimensions (e.g. 20-26). These studies have been widely cited, and form the basis of many influential mass-based paleornithological studies (e.g. 27-29). However, although the variation in allometric datasets can be used to quantify statistically justified upper and lower prediction bounds (e.g. 30,31), many studies have not taken the uncertainty of allometry-based mass predictions into account, instead basing conclusions solely upon a single mean mass estimate (e.g. 18,19,32). This approach is problematic as our inference of mass-dependent biological traits, such as flying ability, can be severely impacted by the variability of body mass estimates (contrast [18,33]). Without constraints on the mass of a putatively flying organism, important aerodynamic parameters such as wing loading cannot be determined [5].

With rare exceptions (e.g. 31), ornithological studies presenting scaling relationships between body mass and skeletal dimensions have not reported prediction intervals associated with their regressions. Prediction intervals depict the standard error of predicted sample means, based on the samples measured in an analysis; this yields a broad, conservative result [34]. In contrast, it is common practice to report confidence intervals for body mass allometries, which only depict the standard error of a regression equation, thereby speciously narrowing the predicted range of body mass estimates [35]. For studies seeking to estimate the live body mass of a fossil organism based on its skeletal dimensions, calculating and applying a prediction interval is therefore crucial to generating robust upper and lower bounds on the mass estimate [36]. 

Here, we present thirteen scaling relationships between body mass and skeletal measurements for extant, flying birds. The analysis covers a wide breadth of extant volant bird diversity, and is among the first extant avian body mass scaling investigations to report prediction intervals for commonly applied allometries (such as femur length and femur circumference). In addition, we compare the precision of these thirteen allometries using both coefficient of determination (R^2^) and percent prediction error (PPE), and assess clade-specific variability in regression parameters in order to assess the relative precision of different correlates, for future use in estimating the body masses of fossil taxa. The 95% prediction intervals associated with the allometries presented here will facilitate the estimation of robust upper and lower bounds on fossil mass estimates, which will be valuable for any future study of mass-dependent characteristics in fossil avialans, such as flight, homeothermy, and encephalization quotients.

## Methods

### Database construction and taxon sampling

**Figure 1:**
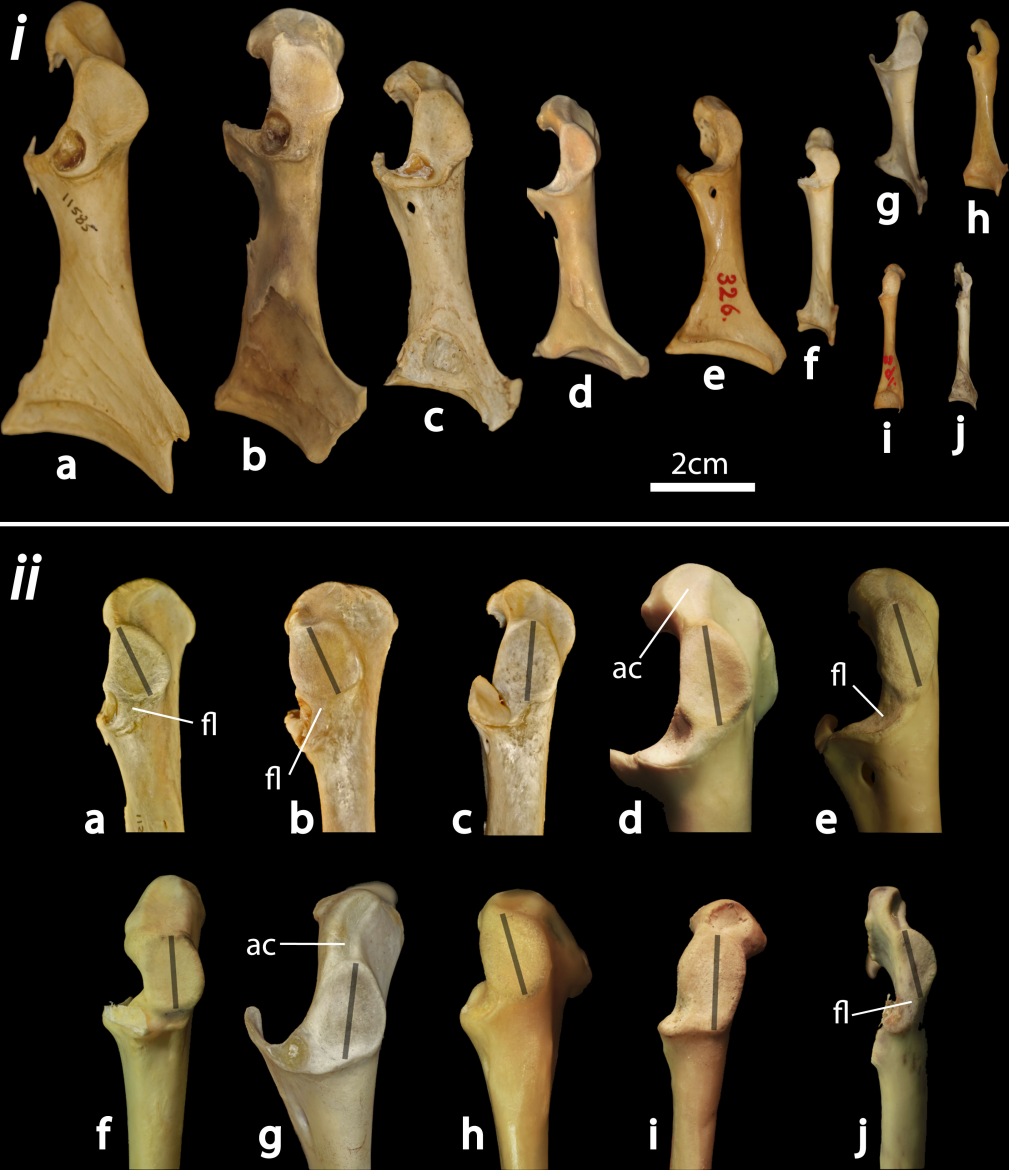
Avian coracoid disparity, and measuring the maximum diameter of the humeral articulation facet. i: Right coracoids in dorsolateral view shown to scale for ten avian taxa with different glenoid geometries. Figured coracoids are a) *Cygnus olor* (Anseriformes: Anatidae), b) *Ardeotis kori* (Gruiformes: Otididae), c) *Balearica regulorum* (Gruiformes: Gruidae), d) *Buteo regalis* (Accipitriformes: Accipitridae), e) *Bubo scandiacus* (Strigiformes: Strigidae), f) *Amazona aestiva* (Psittaciformes: Psittacidae), g) *Puffinus griseus* (Procellariiformes: Procellariidae), h) *Leucophaeus atricilla* (Charadriiformes: Laridae), i) *Podiceps nigricollis* (Podicipediformes: Podicipedidae), and j) *Nucifraga columbiana* (Passeriformes: Corvidae). ii: Close-up of omal coracoid extremities for the taxa listed above (not to scale). Gray lines illustrate the maximum diameter of the HAF. ‘fl’ denotes an extended flange sternolateral to the main portion of the glenoid facet (present in some taxa such as certain anseriforms and gruiforms), which was not included in the HAF measurement. Similarly, ‘ac’ denotes an extended concavity along the acrocoracoid crest (present in some taxa such as certain accipitriforms and procellariiforms), which was not included in the HAF measurement.

863 skeletons of extant, volant birds, with masses spanning three orders of magnitude, were sampled from the Vertebrate Zoology collection of the Yale Peabody Museum (Table S1). To account for sexual dimorphism in body mass estimates, only specimens preserving sex identification data were used. For paired bones, the right side element was measured, unless absent. The following measurements were taken for each specimen: maximum femur length (FL), least femur shaft diameter in anterior view (FD), least femur shaft circumference (FC), maximum humerus length (HL), least humerus shaft circumference (HC), least humerus shaft diameter in anterior view (HD), maximum tibiotarsus length (TiL), maximum tarsometatarsus length (TaL), least tarsometatarsus diameter in anterior view (TaD), least tarsometatarsus shaft circumference (TaC), maximum coracoid lateral length (CLL), least coracoid shaft width (CSW), and maximum diameter of the coracoid’s humeral articulation facet (HAF) (Figure 1). Although tibiotarsus circumference has been demonstrated to scale closely with body mass in birds [23], we found that tibiotarsi were often broken, making any measurement other than maximum length difficult to make precisely. Therefore, this study does not recreate the tibiotarsus regressions presented in [23]. Digital calipers sensitive to 0.01 mm were used for measurements of bone length and diameter, and nylon string was used to measure bone circumference at the point of minimum shaft circumference. For the smallest specimens, circumference was calculated from orthogonal width and depth measurements, after [23]. 

The majority of these measurements have been used as body mass correlates by previous authors (e.g. 20-24,31). In contrast, coracoid HAF dimensions have only been investigated as a potential body mass correlate in one previous study [37], using a relatively small and taxonomically limited dataset. The HAF-bearing portion of the coracoid is amongst the most commonly preserved avian fossil elements in many fossil localities [37-40], underscoring the potential utility of a robust HAF-body mass relationship. Here, we formalized the measurement of avian HAF dimensions by measuring the maximum diameter of the glenoid (Figure 1). Only the main, sub-ovular portion of the glenoid was measured; in instances where a depression extended omal to the main portion of the glenoid onto the acrocoracoid crest (Fig. 1ii d, g), or sternomedial to the main portion of the glenoid as a narrow flange around the scapular cotyla (Figure 1ii a, b, e, j), these extensions were not included in the measurement. A visual depiction of how the measurement was taken for taxa with varying HAF geometries is provided in Figure 1. 

Many previous attempts to estimate the original body masses of fossil taxa suffer due to limited taxonomic sampling of extant taxa, or by ignoring the existence of sexual dimorphism (e.g. 41). To avoid these pitfalls, this study only draws measurements from individuals identified to sex, and samples a greater number of individuals than any previously published avian body mass dataset (see Table S1).

### Statistical Analyses

 The strength of correlation between measured skeletal dimensions and body mass was determined using coefficient of determination (R^2^) values from ordinary least squares regression. Mean body mass estimates and corresponding sex information for each of the bird species were obtained from [42]. In order to investigate the differences between regressions for mean body mass and actual body mass, we compared regressions between the maximum diameter of the coracoid HAF, and both mean and recorded individual body mass for a subset of our total dataset. This subset, comprising the only specimens associated with recorded body mass in our dataset, represented 115 individuals, from 31 genera in 9 avian orders. Scaling relationships and strength of correlation were compared using standardized major axis (SMA) regression using the package Smatr in R [43]. For all analyses, we used the natural log (ln) of these data to mitigate the effects of extreme outliers on regression coefficients [30,34]. 

We subsequently used regression equations for each measured skeletal correlate to predict body mass, and calculated percent prediction error (PPE) by comparing predicted with observed body mass in the style of [30]. Because clade- and ecology-specific variations in gait and limb posture may subject the vertebrate skeleton to different stress regimes (e.g. 23,30), the strength of various skeletal measurements as body mass correlates might be expected to vary among taxonomic groups. We therefore repeated our analyses for the entire dataset broken down into individual avian orders. All analyses were performed using the statistical software R version 2.15.2 [43]. 

## Results

**Figure 2:**
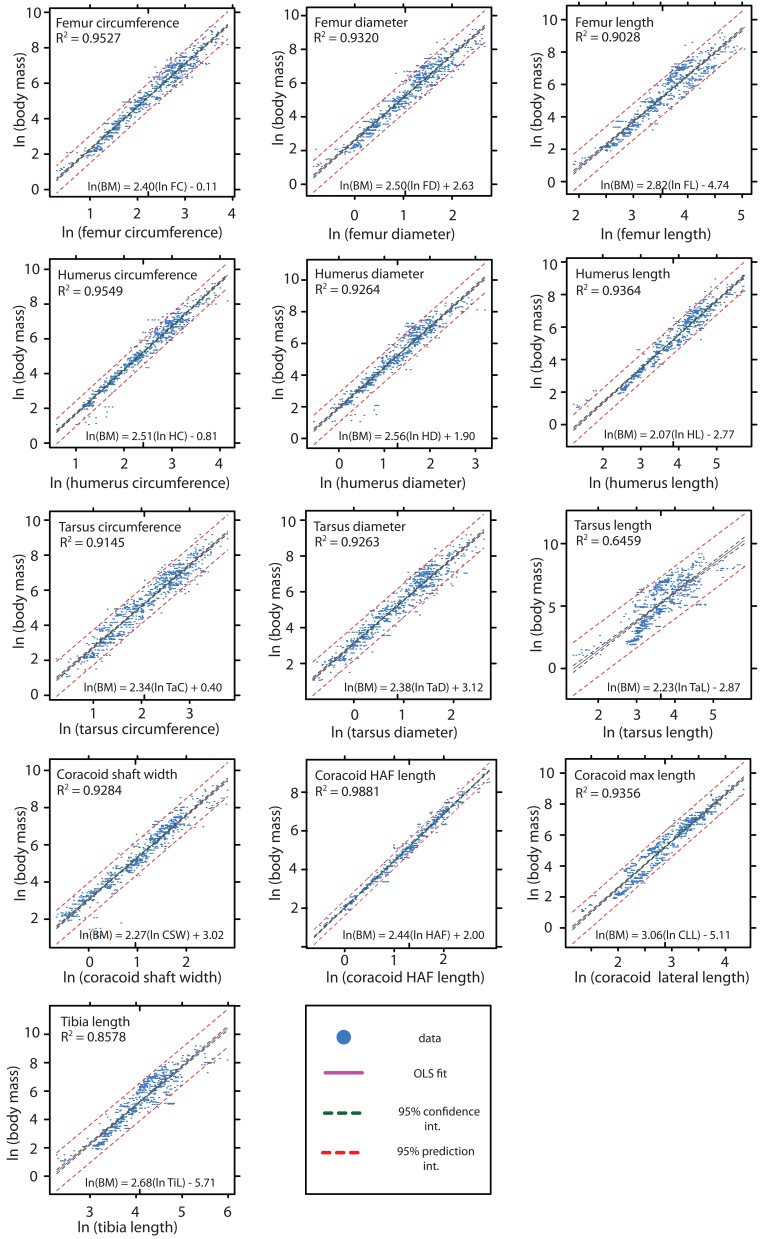
Ordinary least squares regressions between ln body mass and ln skeletal element dimensions in a sample of 863 volant birds. Regression equations are shown in the format *y* = *mx* +*b*, and presented with their coefficient of determination (*R* ^2^) in panels. 95% prediction intervals (red), and 95% confidence intervals (green) are also depicted. At this scale, 95% confidence intervals virtually overlap the OLS fit (pink) for the more precise regressions. Regression statistics are presented in Table 2.

**Figure 3:**
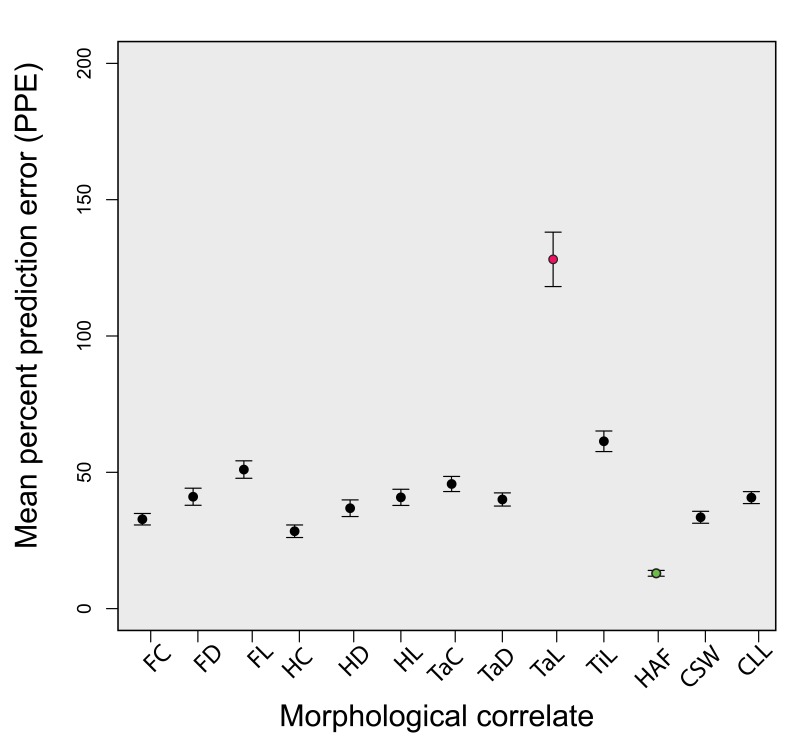
Comparison of the predictive power of several body mass proxies based on their mean percent prediction error (PPE). Mean PPE for each correlate is represented by a circle, with 95% confidence intervals in black. The least precise correlate as reckoned by PPE (tarsometatarsus length) is shown in red, while the most precise (coracoid HAF length) is shown in green.

**Figure 4:**
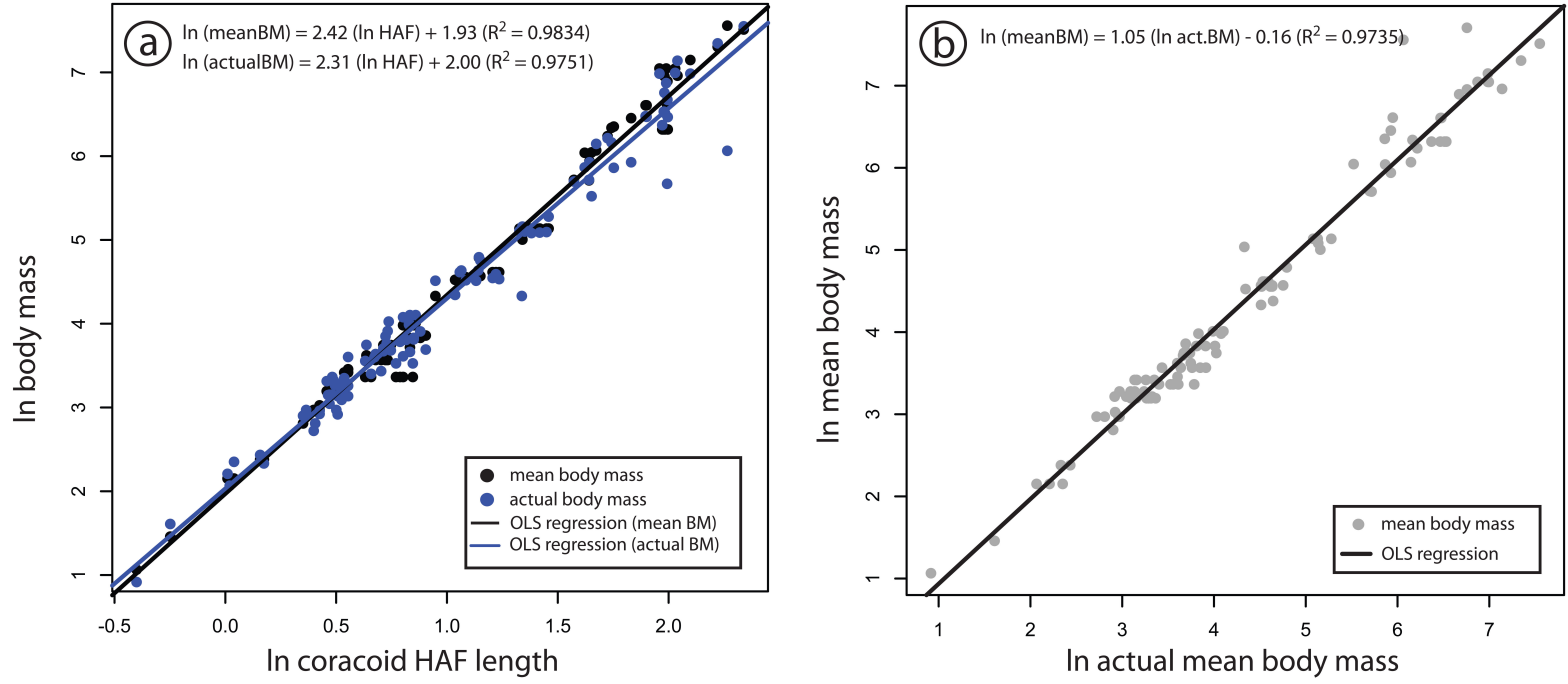
Scatterplot illustrating close correspondence of SMA regressions between coracoid HAF and mean species body mass (a), and coracoid HAF and recorded individual body mass (b). Coefficient of determination and equations (in format *y* = *mx* +*b*) are given in panels. Statistics for comparison via SMA regression are given in Table 2.

Within the combined dataset, all of the studied skeletal dimensions show moderate- to high correlation and linear fit with body mass; all R^2^ values fall between 0.6459 and 0.9881, and only one (tarsus length) falls below 0.85 (Figure 2; Table 1). The length of the coracoid HAF represents the most precise overall scaling relationship in our analysis (R^2^ = 0.9881). The mean percent prediction error (PPE) for these regressions typically falls in the range of 10-60% (Figure 3; Table 1), with the least precise correlate being tarsus length (mean PPE = 128.11%), and the most precise again being the coracoid’s HAF (mean PPE = 12.95%). Sex, age, and life history can all have a significant impact on the body mass of individual birds [23], such that the body mass of individuals may differ significantly from recorded mean masses for avian species. We therefore tested the robustness of our result for coracoid HAF length comparing the strength of correlation between HAF length and mean species body mass, and HAF length and recorded individual body mass, for a subsample of our total dataset (n=115) using standardized major axis (SMA) regression. This subsampled dataset comprises 44 species, representing 9 avian orders (see Table 1). The results of SMA regression reveal extremely similar slopes, intercepts, and coefficients of determination for regressions using mean body mass for species, and actual body mass of individuals (Figure 4); 95% confidence intervals calculated around slopes and intercepts between these two regressions overlap (Table 2). This suggests that both mean species body mass and recorded individual body masses are indistinguishable in terms of their strength and utility as body mass correlates in this large, taxonomically diverse dataset.

**Table 1:** Coefficient of determination, slopes, intercepts, upper and lower 95% prediction intervals, and mean PPE values for all regressions (combined dataset) between body mass and 13 skeletal dimensions.

	FC	FD	FL	HC	HD	HL	TaC	TaD	TaL	TiL	HAF	CSW	CLL
Slope	2.4	2.5	2.82	2.51	2.56	2.07	2.34	2.38	2.23	2.68	2.44	2.27	3.06
y-Intercept	-0.11	2.63	-4.74	-0.81	1.9	-2.77	0.4	3.12	-2.87	-5.71	2	3.02	-5.11
R2	0.95	0.93	0.9	0.95	0.93	0.94	0.91	0.93	0.65	0.86	0.99	0.93	0.94
Upper 95% Prediction Interval	0.76	0.92	1.11	0.75	0.95	0.89	1.03	0.95	2.11	1.34	0.38	0.94	0.9
Lower 95% Prediction Interval	-0.76	-0.92	-1.11	-0.75	-0.95	-0.89	-1.03	-0.95	-2.11	-1.34	-0.38	-0.94	-0.9
Mean PPE	32.79	41.04	51.01	28.38	36.83	40.81	45.72	40.04	128.11	61.38	12.95	33.51	40.72
PPE 95% CI	2.11	3.13	3.2	2.3	3.05	2.96	2.77	2.42	9.98	3.77	1.07	2.19	2.2

Body mass is taken as sex-specific means for individual species (from Dunning, 2007).

**Table 2:** Results (coefficient of determination, slopes, intercepts, and corresponding 95% confidence intervals) of SMA regression comparing the correlations between coracoid HAF vs mean body mass (from Dunning, 2007), and coracoid HAF vs recorded individual body mass.

	R2	Slope	Slope 95% CI (lower bound)	Slope 95% CI (upper bound)	Y-intercept	Y-intercept 95% CI (lower bound)	Y-intercept 95% CI (upper bound)
Mean Body Mass (g)	0.9834	2.42	2.36	2.48	1.93	1.87	2
Actual Body Mass (g)	0.9751	2.31	2.24	2.38	2	1.92	2.08

Note that CIs of both slope and intercept overlap.

Broken down by avian order, results of ordinary least squares regression and PPE demonstrate significant differences among taxonomic groups, both in terms of the overall strength of correlation between the measured skeletal elements and body mass, and in terms of the relative strength of correlation among different skeletal elements. Coefficients of determination between our skeletal correlates and body mass, partitioned by avian order, are summarized in Table 3; the slopes, intercepts, corresponding p-values, and prediction intervals for these regressions are given as supplementary information (Tables S2, S3, S4, S5, S6, S7). PPE plots for treated avian orders are given in Figure S1. 

**Table 3:** Coefficient of determination for regressions between (mean) body mass and 13 skeletal measurements, for 18 avian orders.

	FC	FD	FL	HC	HD	HL	TaC	TaD	TaL	TiL	HAF	CSW	CLL
Accipitriformes	0.8306	0.8884	0.8404	0.8803	0.8431	0.9353	0.7148	0.7572	0.5272	0.7865	0.9939	0.8301	0.9106
Anseriformes	0.8253	0.7867	0.9162	0.7826	0.6729	0.8773	0.7153	0.7867	0.8116	0.8879	0.9337	0.8796	0.91
Apodiformes	0.9368	0.898	0.9561	0.6864	0.7063	0.9534	0.6434	0.8093	0.8082	0.9238	0.9878	0.7109	0.9577
Charadriiformes	0.9092	0.9105	0.9346	0.9631	0.92	0.9508	0.9063	0.8948	0.3206	0.6147	0.9744	0.9502	0.9804
Ciconiiformes	0.9171	0.8671	0.8422	0.9293	0.8937	0.9188	0.8914	0.7748	0.8471	0.8757	0.9625	0.806	0.8958
Columbiformes	0.9026	0.8929	0.8971	0.8618	0.7596	0.889	0.7324	0.725	0.7696	0.9047	0.9991	0.8837	0.9148
Coraciiformes	0.9707	0.979	0.9828	0.9814	0.9656	0.9658	0.9013	0.9149	0.5854	0.9643	0.9836	0.9786	0.9644
Falconiformes	0.9163	0.9144	0.9637	0.8051	0.6961	0.954	0.8525	0.9129	0.7886	0.9433	0.9987	0.7491	0.9323
Galliformes	0.9253	0.9472	0.9164	0.9304	0.8577	0.7491	0.7481	0.8676	0.7634	0.8317	0.9258	0.8896	0.9538
Gruiformes	0.9634	0.9369	0.8577	0.9443	0.8744	0.9181	0.9045	0.8924	0.8105	0.8761	0.9655	0.876	0.9357
Passeriformes	0.9454	0.9306	0.9462	0.9529	0.9313	0.9771	0.8824	0.8907	0.8715	0.9117	0.9864	0.9207	0.9688
Podicipediformes	0.7933	0.8966	0.8819	0.8064	0.4355	0.9634	0.7603	0.6788	0.9318	0.9465	0.967	0.9707	0.9394
Procellariiformes	0.9593	0.9515	0.9905	0.98	0.9771	0.9728	0.951	0.9582	0.7126	0.8425	0.983	0.97	0.9848
Psittaciformes	0.8746	0.9339	0.9619	0.9294	0.8495	0.983	0.8567	0.8312	0.7429	0.9447	0.9716	0.9397	0.9616
Strigiformes	0.8932	0.9183	0.9233	0.9007	0.8498	0.8901	0.8373	0.9162	0.4264	0.7783	0.9169	0.8989	0.902
Suliformes	0.8666	0.9235	0.938	0.9003	0.868	0.934	0.8882	0.8828	0.9589	0.971	0.961	0.9019	0.9735
Trogoniformes	0.7898	0.6046	0.9851	0.666	0.9191	0.9893	0.8764	0.818	0.9925	0.8997	0.994	0.9314	0.9548
Caprimulgiformes	0.3847	0.6884	0.8788	0.1423	0.7487	0.9472	0.797	0.6456	0.1005	0.3562	0.8337	0.634	0.7761

**Figure 5:**
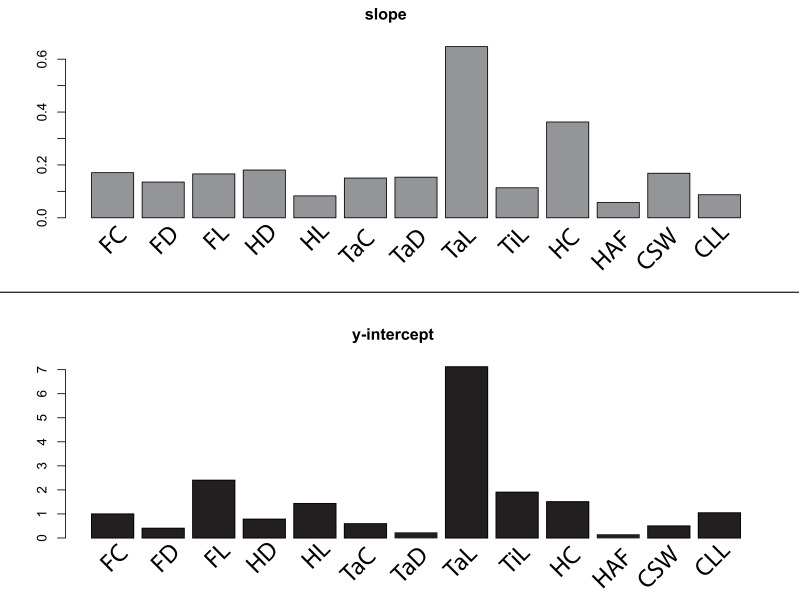
Barplots illustrating variance in slopes (gray) and intercepts (black) of regressions between body mass and our 13 skeletal measurements, among all treated avian orders.

The relative ordering of measured skeletal elements in terms of their strength as body mass correlates varies widely among taxonomic groups: correlation coefficients are highest between body mass and coracoid HAF length in 10 of the 18 treated avian orders (Accipitriformes, Anseriformes, Apodiformes, Ciconiiformes, Columbiformes, Coraciiformes, Falconiformes, Gruiformes, Passeriformes, and Trogoniformes). In Charadriiformes, Galliformes, and Pelecaniformes, maximum coracoid lateral length provides the strongest correlation with body mass. Femur length is the strongest body mass correlate in Procellariiformes and Strigiformes, humerus length exhibited the highest correlation coefficient in Psittaciformes and Caprimulgiformes, and coracoid shaft width was the most precise body mass correlate for Podicipediformes. In order to quantify the relative utility of our measured skeletal elements across different avian clades, we calculated the variance in regression slopes and intercepts for each element among all avian orders (illustrated as barplots in Figure 5); high variance indicates that the strength of a specific skeletal element as a potential body mass correlate is highly taxon-specific, and should therefore be treated with caution. Alternatively, low variance indicates that the strength of correlation is largely independent of taxonomic affinity, as well as potentially confounding ecological variables such as flight style and habitat preference. Our data demonstrate that variance in both regression slope and intercept among avian orders is lowest for coracoid HAF length, and highest for tarsus length. Femur length, humerus length, and tibia length also show elevated variance in slopes and/or intercepts among orders, and therefore may constitute relatively unreliable indicators of original body mass for fossil birds.

## Discussion

### Ecology and clade-dependence of body mass regressions

Our data illustrate several important aspects of allometric scaling relationships among 18 orders of extant, volant birds: the strength of our 13 measured body mass correlates differ significantly overall, and the relative precision of different elements as body mass predictors sometimes varies significantly among broad taxonomic groups, likely reflecting adaptive ecological overprinting in clades exhibiting widely disparate ecologies [44]. However, the high precision of many of the correlations presented here indicates that, regardless of potentially confounding factors such as phylogenetic position, ecology, and flight style, these regressions can be used to infer relatively narrow upper and lower bound prediction intervals on body mass estimates across Aves, as well as for fossil flying birds (Figure 2). These findings are consistent with several previous studies that have investigated the dimensions of avian skeletal elements as potential body mass correlates [20-24]. However, we demonstrate that among the skeletal elements examined here, the maximum diameter of the coracoid HAF is generally the most accurate predictor of avian body mass, and that the slopes and intercepts of this regression among all studied avian orders show the least variance. These findings suggest low ecological overprinting of HAF dimensions in extant flying birds, and illustrate this measurement’s potential utility for the accurate estimation of body mass in fossil flying birds. In addition, these analyses show that the coracoid HAF serves as an equally good predictor of body mass when either mean species body mass, or recorded individual body mass is considered. We therefore suggest that in instances where close correspondence between mean species and true body masses can be demonstrated (Figure 4), using mean species body masses in allometric studies will enable more specimens, and a correspondingly broader taxonomic sample to be investigated. 

We also acknowledge two additional factors affecting our data and analyses that may have an effect on the final regression parameters: 1) these data are not strictly independent, as the 863 studied specimens exhibit varying degrees of common ancestry, which may spuriously increase or decrease the strength of allometric scaling relationships. In addition, 2) our use of multiple individuals belonging the same species in the dataset may also skew our calculated regression parameters. Although 1) can be addressed by employing generalized least squares models (e.g. 45), the low interordinal variance exhibited by many of the regressions presented here (Figure 5, Table 4) indicates relatively little phylogenetic dependence in this dataset. Previous allometric studies have demonstrated largely insignificant phylogenetic dependence of limb scaling patterns in terrestrial quadrupeds [30], and inappropriate application of phylogenetic regression can result in poor statistical performance [46]. Further, our broad taxonomic sampling across Aves complicates the acquisition of a robust phylogeny with a specified set of branch lengths incorporating every taxon in this analysis, which would be beyond the scope of this study. For these reasons, and for ease of comparison with previously published avian allometric studies [30], we have elected to present OLS results instead of phylogenetic regressions. Future work may delve into the question of phylogenetic correlation in avian allometric data.

**Table 4:** Calculated variance in slopes and intercepts of regressions between body mass and our 13 skeletal measurements, for 18 avian orders.

	FC	FD	FL	HC	HD	HL	TaC	TaD	TaL	TiL	HAF	CSW	CLL
Y-Intercept Variance	1	0.4	2.4	1.51	0.78	1.43	0.59	0.22	7.12	1.9	0.14	0.5	1.05
Slope Variance	0.17	0.14	0.17	0.36	0.18	0.08	0.15	0.15	0.65	0.11	0.06	0.17	0.09

We attempted to address 2) by re-running analyses on two restricted datasets where multiple specimens of any single species were omitted by random deletion. We then compared the relative ordering of skeletal measurements as body mass correlates, both as coefficient of determination and PPE, with those of our original analyses. Sample sizes for these single-individual analyses were 317 (the number of individual species in our dataset). Regression parameters and coefficients of determination resulting from analysis of these restricted datasets were extremely similar to those of the total dataset (Table S8), and the re-analysis resulted in minimal re-ordering of skeletal measurements in terms of their strength as body mass predictors (Figure S2).

The varying strengths of our skeletal measurements as body mass correlates, both within the entire dataset, and among different avian orders, probably reflect the influence of ecological and biomechanical constraints on element dimensions. Although the ecological and phylogenetic importance of many of these data will be discussed elsewhere, we highlight some of the broader patterns concerning the strength of the coracoid HAF as a morphological correlate for body mass, in a comparative context with other frequently applied skeletal measurements. In particular, the strength of the observed correlation between coracoid HAF dimensions and body mass likely has its basis in flight biomechanics; failure of the shoulder joint in a wild flying bird would probably be fatal, and larger birds appear to exhibit larger shoulder articulations to provide adequate safety factors for stress dissipation during the active flight stroke. Additionally, the circumferences of hindlimb elements scale closely with body mass, as these bones are subject to numerous biomechanical and energetic trade-offs, and as such have narrow tolerance limits (see 21,23,47-50). 

Conversely, the dimensions of some avian skeletal elements can be more heavily influenced by ecological, as opposed to biomechanical factors, such as wading and prey handling [51]. Such elements are consequently expected to make poor body mass correlates. We suggest that the relatively weak correlation found between tarsus length and body mass reflects one such example of ecological overprinting; certain ecological groups (i.e., waders) exhibit disproportionately long tarsometatarsi, while others (i.e., raptors and owls) tend to have disproportionately short tarsometatarsi—a specialization related to prey handling efficiency [51,52]. In support of this, the variance in regression parameters for tarsometatarsus length among the 18 avian orders sampled here vastly exceeds that of any other regression parameter (Figure 4; Table 4). These analyses demonstrate that skeletal measurements exhibiting high interordinal variance would likely comprise poor predictors of body mass in fossil birds, unless the fossil in question could be confidently diagnosed to the crown-group of an order represented in our sample. 

Coracoid dimensions have been used to estimate body mass for fossil birds previously (e.g. 31,37,53). In particular, Elzanowski et al. (2012) [31] generated several highly precise coracoid-based allometries for mass estimation of fossil Procellariiformes. Two of these correlates (minimum coracoid shaft width and maximum lateral length of the coracoid) are examined in the present study. Although the correlation coefficients reported by Elzanowski et al. (2012) [31] are higher than those reported here, in our dataset Procellariiformes show the highest overall body mass correlation of all studied orders (mean correlation coefficient of all allometries, see Table 3), indicating that a wide variety of skeletal measurements may provide accurate body mass estimates in this clade. Although the regressions for minimum coracoid shaft width and maximum lateral coracoid length exhibit relatively low interordinal variance overall (Figure 5), they are surpassed by other correlates as preferable predictors of avian body mass (Figure 2).

**Figure 6:**
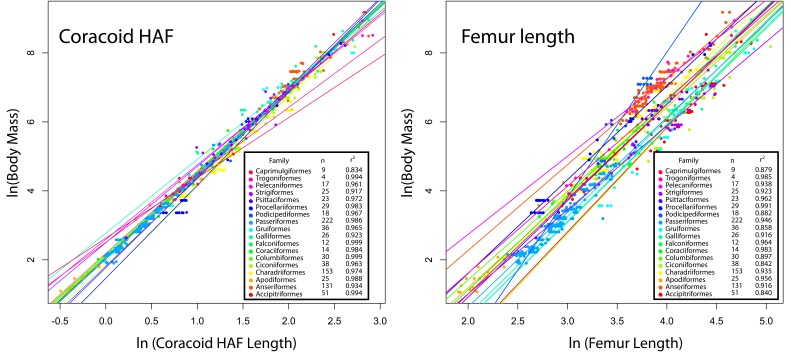
Scatterplots illustrating the variance in OLS regression lines for 18 avian orders, comparing (left panel) body mass vs coracoid HAF, and (right panel) body mass vs femur length. Corresponding sample sizes and coefficients of determination are given in plots.

One of the most frequently used skeletal measurements for estimating body mass in fossil crown- and stem-birds is femur length (e.g. 13,14,54); here we show that the interordinal variance in slope and intercepts of regression between femur length and body mass is substantially higher than in the length of the coracoid HAF (Figure 6). The much tighter clustering in OLS regression lines indicates greater reliability in using coracoid HAF rather than femur length when estimating body mass across a variety of taxonomic groups. As such, we suggest that a variety of other body mass correlates, including maximum HAF diameter and maximum coracoid lateral length be used in lieu of femur length for estimating body mass in fossil volant birds, when these elements are preserved (Figure 2). 

Scaling relationships and allometry are widely accepted as a useful means to estimate body mass for fossil crown-group taxa [14,30,55]. However, the applicability of such methods for estimating body mass in more distantly related stem-group taxa, especially those that fall outside the body size range observed in extant clade representatives, is less well understood ([30], references therein). As a result, we cannot presently justify applying these extant avian body mass regressions to distantly related stem taxa (for example to basal theropods); however, crown group scaling relationships such as the ones described here are currently the most robust method for estimating mass in crownward stem taxa falling within the range of body masses in the extant dataset (such as *Ichthyornis*) [30]. Estimation of body mass with associated prediction intervals in these presumably volant stem-group taxa will provide a level of robustness to body mass-dependent paleobiological studies that has been elusive up to this point. 

### Paleobiological implications

Addressing many of the fundamental biological and ecological questions surrounding the early evolution of modern birds requires fully articulated (and thus exceptional) fossils. However, the lightweight, buoyant, and relatively fragile nature of bird skeletal remains conspires against their articulated preservation in many depositional settings [56-60]. As such, complete bird fossils are rare, and often make up a disproportionately small percentage of vertebrate remains from otherwise well sampled, fossiliferous localities [61]. However, elements comprising the avian pectoral girdle, in particular the omal extremity of the coracoid, are represented in many assemblages disproportionately often, with humeri, tarsometatarsi, and tibiotarsi also being commonly preserved (e.g. 37,38,57). A scientific premium is therefore placed on discovering new physiological and ecological correlates (such as those afforded by the allometric scaling relationships described here) that can extract additional information from this largely fragmentary and depauperate fossil record. 

In addition, although isolated, fragmentary fossil bird bones are often of limited taxonomic use, several recent studies have revealed that the omal extremity of the coracoid can be phylogenetically informative. In fact, the avian coracoid is amongst the richest sources of characters for cladistic analyses of fossil birds [37,62]. The phylogenetic utility of omal coracoid extremities was illustrated by Longrich et al. (2011) [37], who discovered definitive evidence for avian mass extinction at the K-Pg boundary solely on the basis of coracoid morphotypes present above and below the boundary. As such, establishing coracoid measurements as robust body mass proxies holds valuable paleobiological implications: a single, isolated omal coracoid fragment can theoretically be used to diagnose taxonomic affinity, body mass, and by extension, estimates of a host of additional macroecological and biomechanical attributes such as a species’ energetic demands, maximum population density, and aspects of locomotory mechanics [5,7,8,63]. 

## Conclusions

 In summary, the most broadly useful body mass correlates for fossil taxa are those that are simple to quantify, statistically robust, and commonly preserved in the fossil record. These correlates will also be those where the vagaries of phylogenetic history, gait, and limb posture do not substantively influence the body mass regression [30]. Here, we have quantified the statistical precision of several regressions that appear to meet these criteria for volant birds, and present several correlates that scale more precisely with body mass than does femur length (a commonly applied body mass correlate). The results of both least squares regression and PPE demonstrate that the maximum diameter of the coracoid HAF is the most precise predictor of volant avian body mass in this dataset. Although all measured elements (with the exception of tarsus length, which emerges as a significant outlier) perform surprisingly well, and have been used in previous studies to estimate the body mass of extinct avian taxa, the HAF measurement performs significantly better in these analyses (Figures 2-3), and should be considered as a potential body mass estimator when it is preserved. We also provide a measure of taxonomic group- and ecology-dependence in skeletal body mass correlates, by analyzing the variance in slope and intercept of ordinary least squares regression across 18 avian orders, and demonstrate that coracoid HAF length shows the least variance of any measured skeletal element in this dataset. The maximum diameter of the coracoid HAF therefore appears to constitute the most precise correlate for estimating body mass in this dataset, and exhibits the least variance amongst different avian orders. As such, we suggest that, for volant birds, the relationship between maximum coracoid HAF dimensions and body mass is largely independent of phylogenetic relationships and potential ecological overprinting. 

All of the regressions presented here will prove useful for the robust estimation of body mass in extinct volant avialans, by facilitating the estimation of statistically justified upper and lower bounds on fossil body mass estimates. These data stand to become a valuable resource for a variety of paleobiological investigations on topics ranging from the origin of avian flight [64], to meta-analyses of avian body mass evolution. 

## Supporting Information

Figure S1 **Mean PPE plots with 95% confidence intervals for 18 avian orders and 13 skeletal measurements.**
(PDF)

Figure S2 **Mean PPE plots with 95% prediction intervals for 317 species (one specimen per species).** The total dataset (317 species, 863 specimens) was pruned to 317 specimens using two random partitions. The relative ordering of body mass correlates is virtually unchanged in both cases from the total dataset.
(PDF)

Table S1 **Limb measurements and body mass data.**
(CSV)

Table S2 **Slopes (m) for regressions between (mean) body mass and 13 skeletal measurements, for all avian orders.**
(CSV)

Table S3 **Slope p-values for regressions between (mean) body mass and 13 skeletal measurements, for all avian orders.**
(CSV)

Table S4 **Intercepts (b) for regressions between (mean) body mass and 13 skeletal measurements, for all avian orders.**
(CSV)

Table S5 **Intercept p-values for regressions between (mean) body mass and 13 skeletal measurements, for all avian orders.**
(CSV)

Table S6 **Prediction intervals for regressions between (mean) body mass and 13 skeletal measurements, for all avian orders.**
(XLSX)

Table S7 **Mean PPE for regressions between (mean) body mass and 13 skeletal measurements, for all avian orders.**
(CSV)

Table S8 **All regression parameters for two pruned datasets of 317 species (one individual per species).**
(CSV)
