# Supplementary figures and images for: Skeletal Correlates for Body Mass Estimation in Modern and Fossil Flying Birds
Source: PLoS One. 2013 Nov 29;8(11):e82000. doi: 10.1371/journal.pone.0082000 (PMC3843728; doi:10.1371/journal.pone.0082000)

1

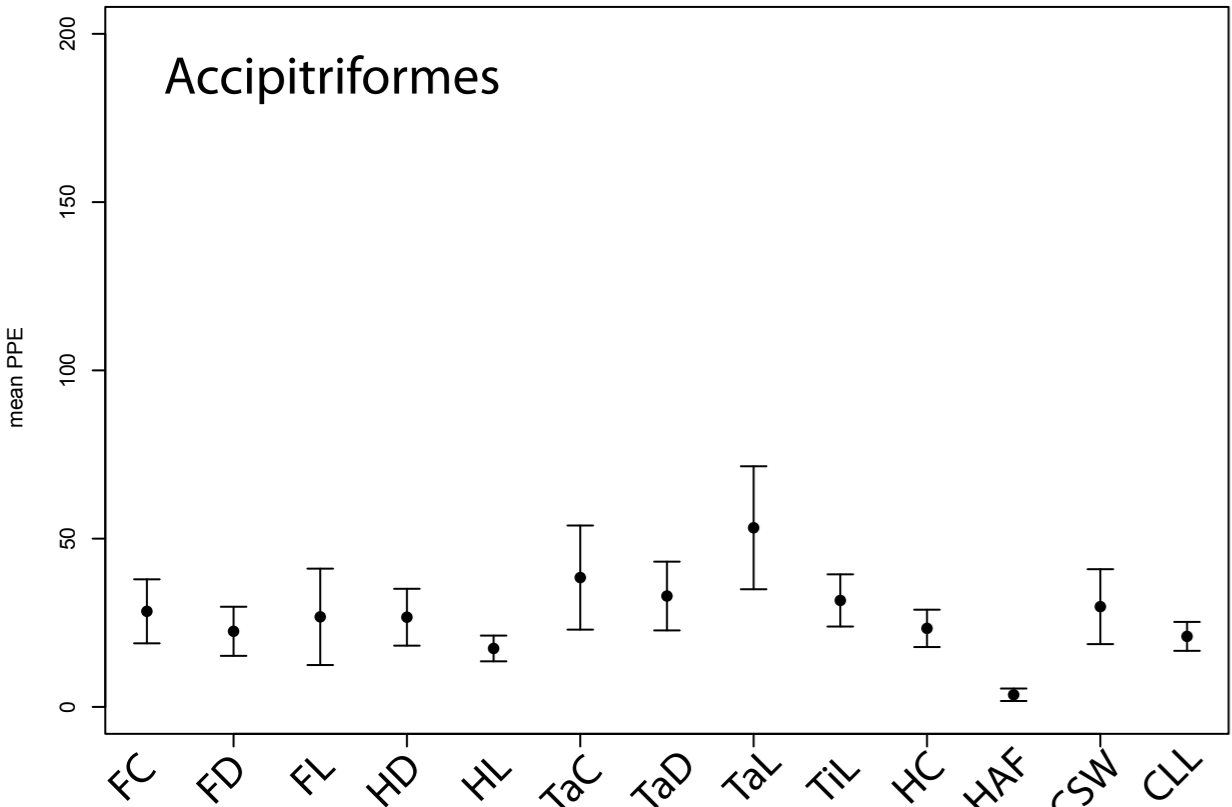

2

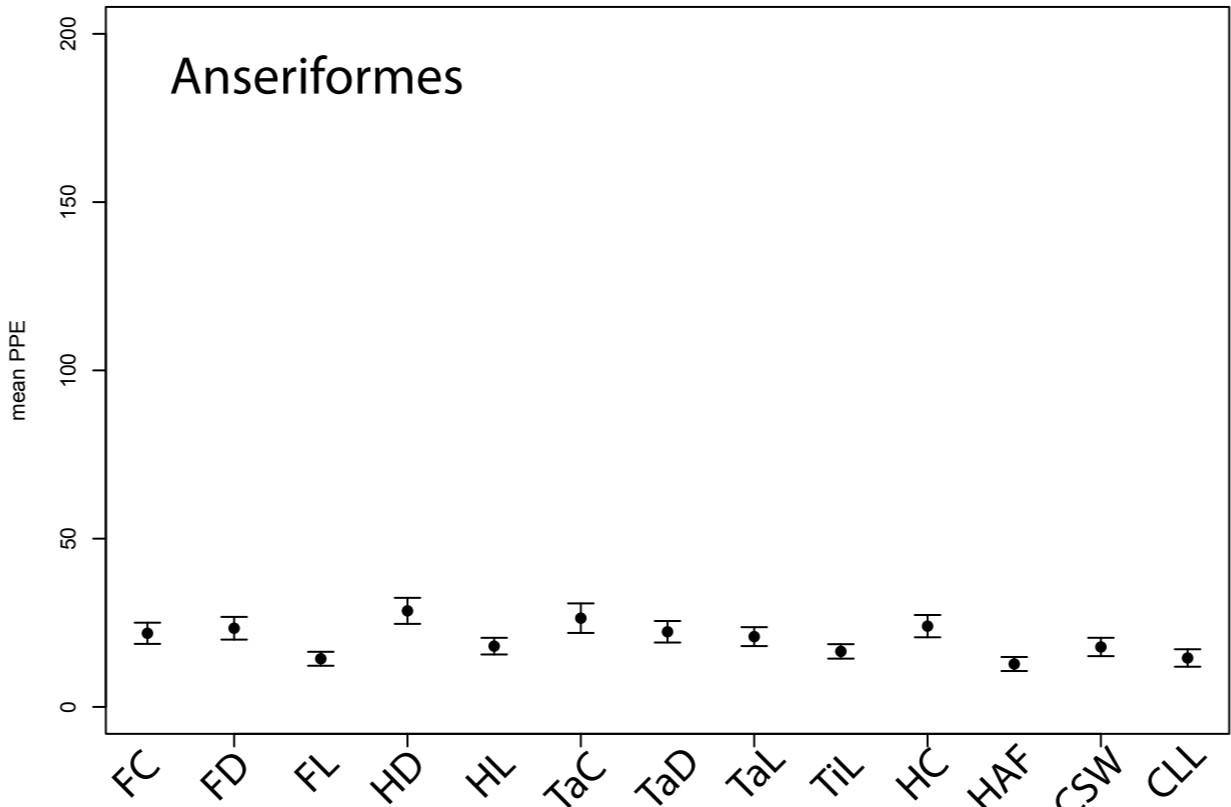

3

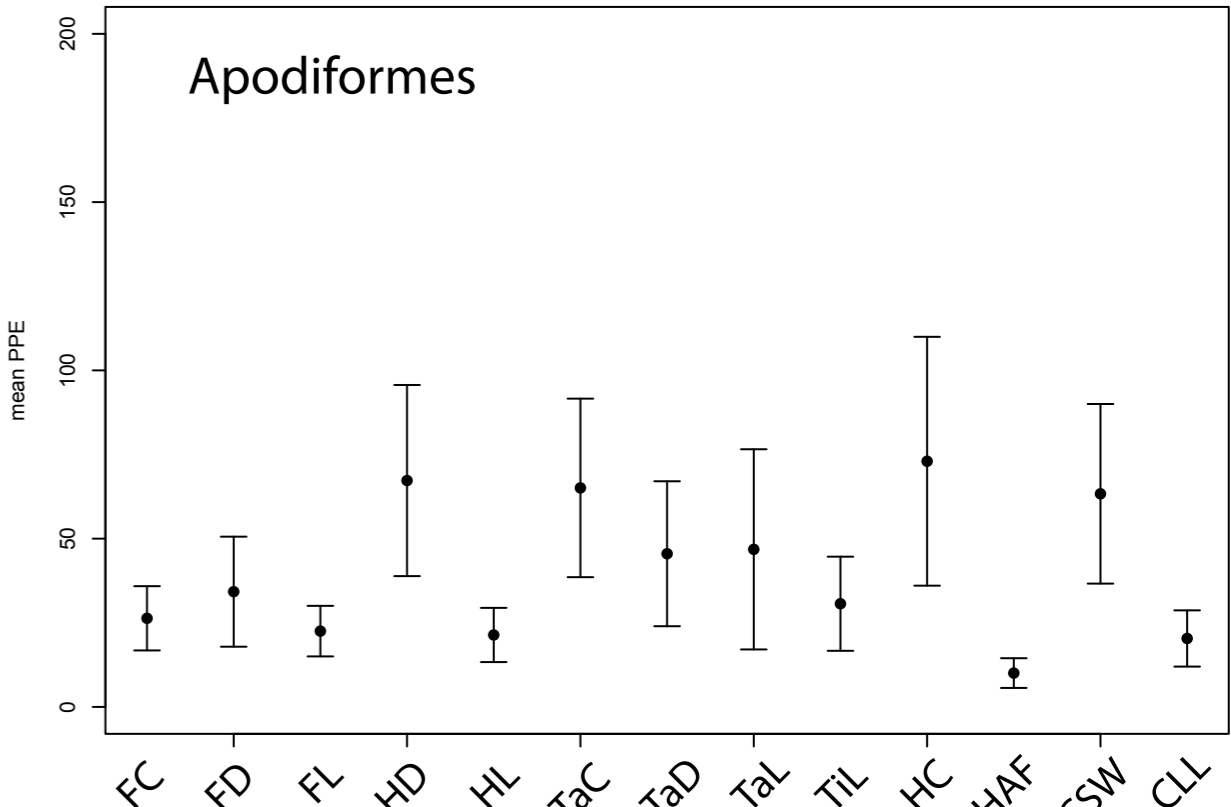

4

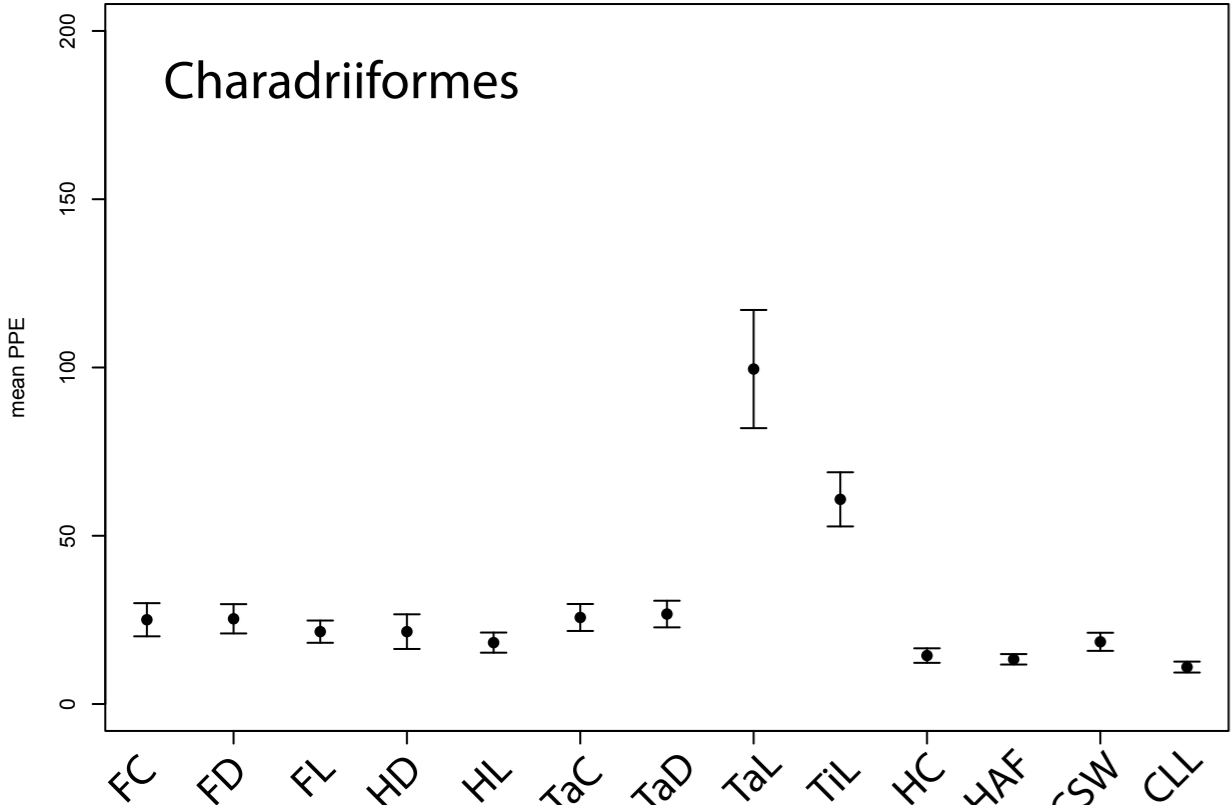

5

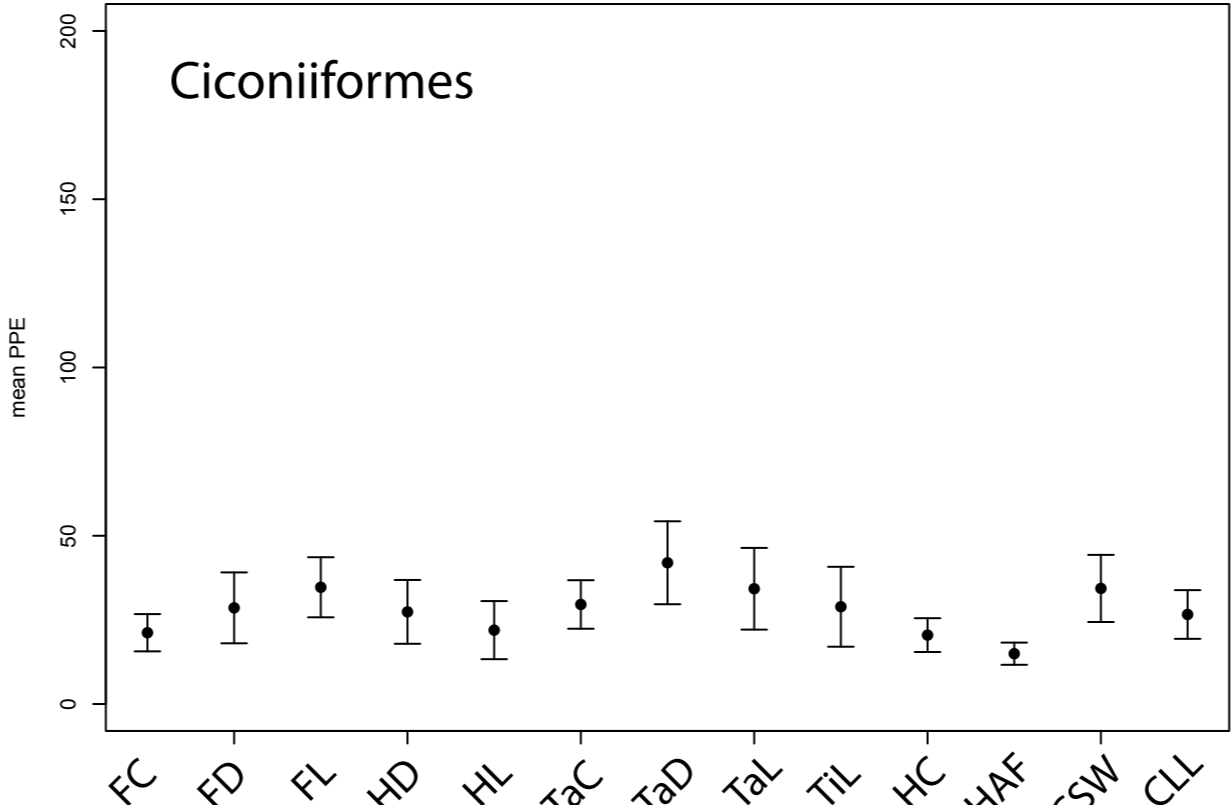

6

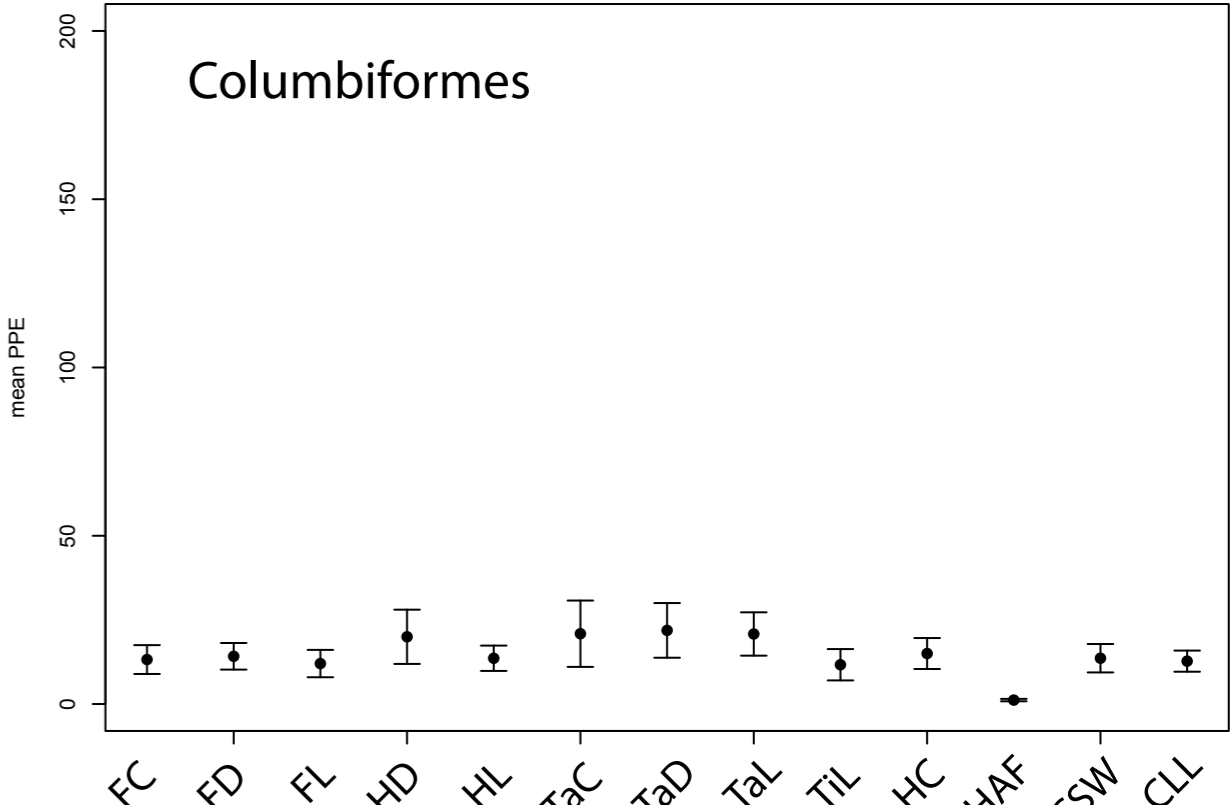

7

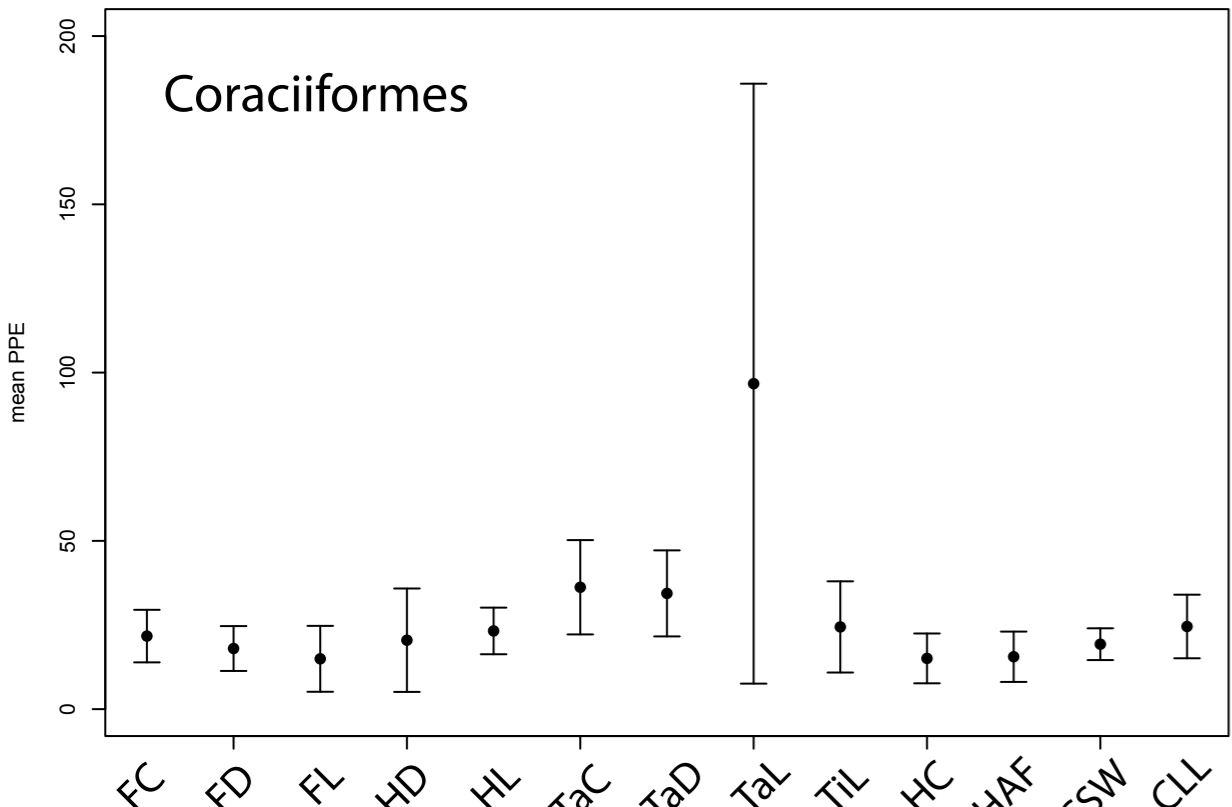

8

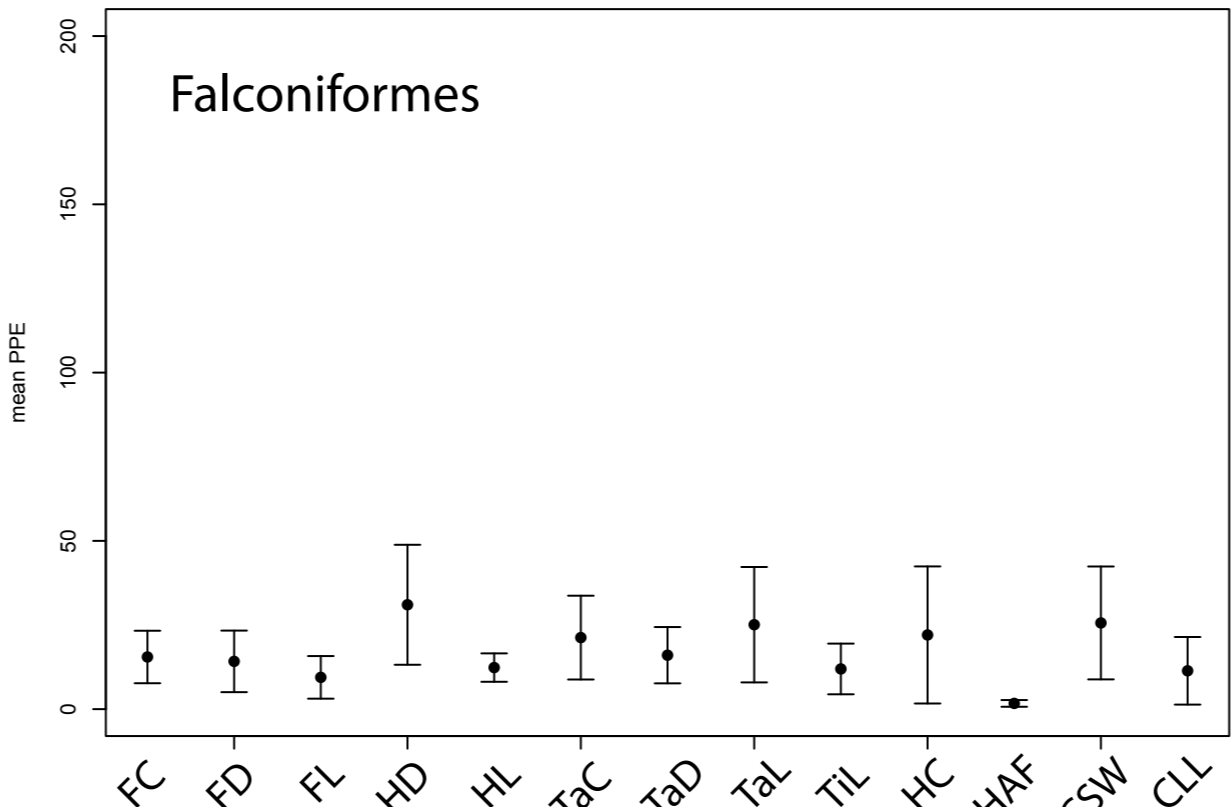

9

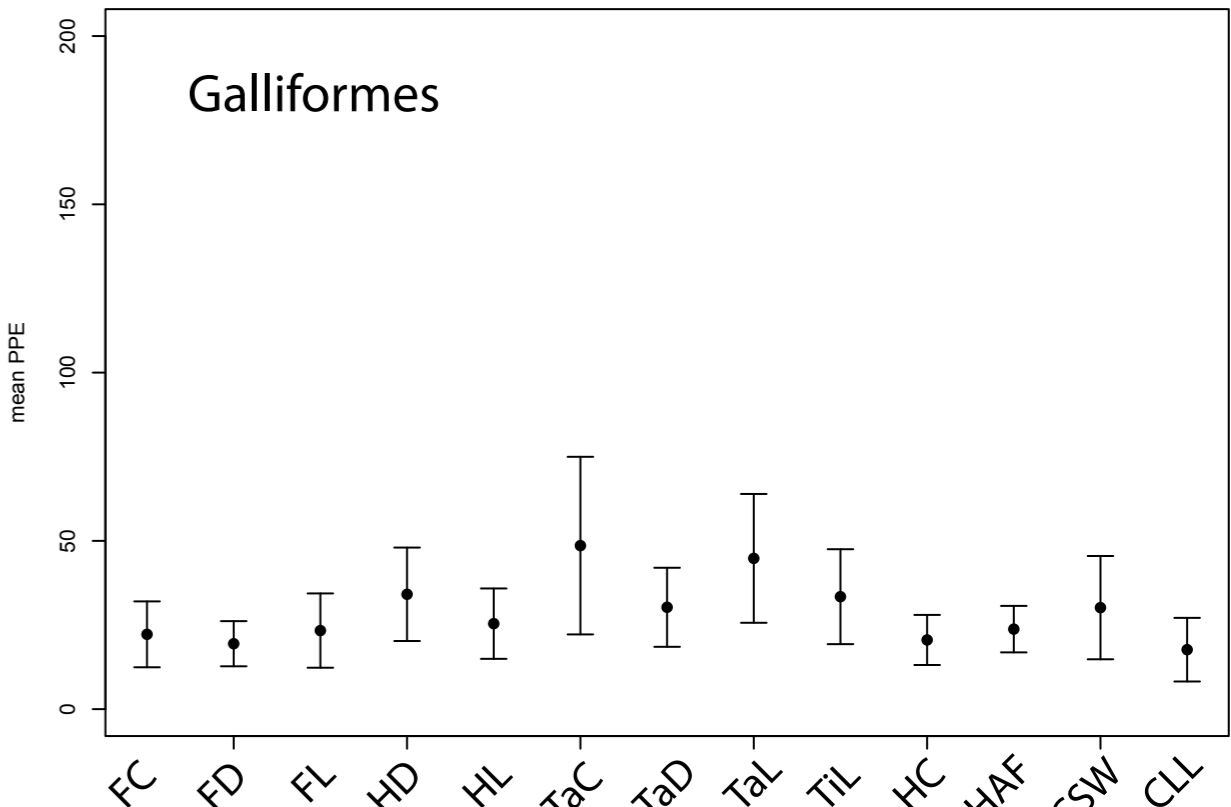

10

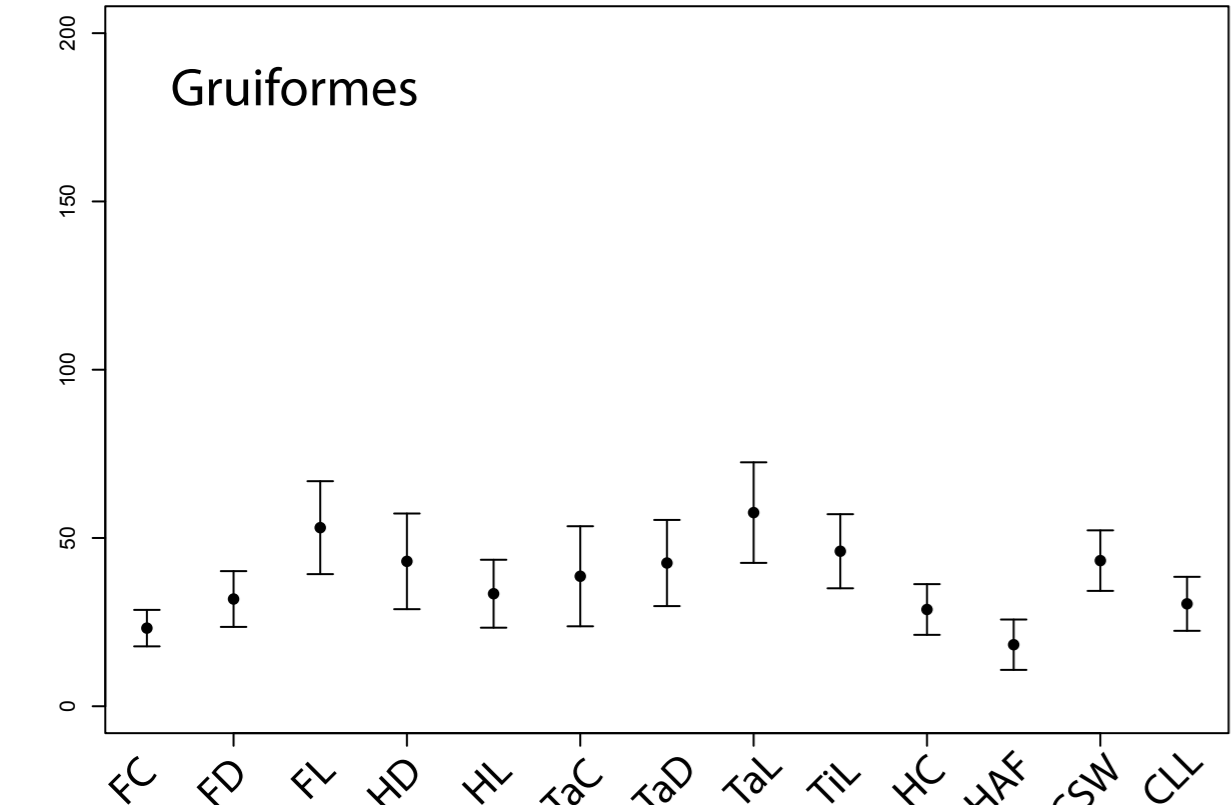

11

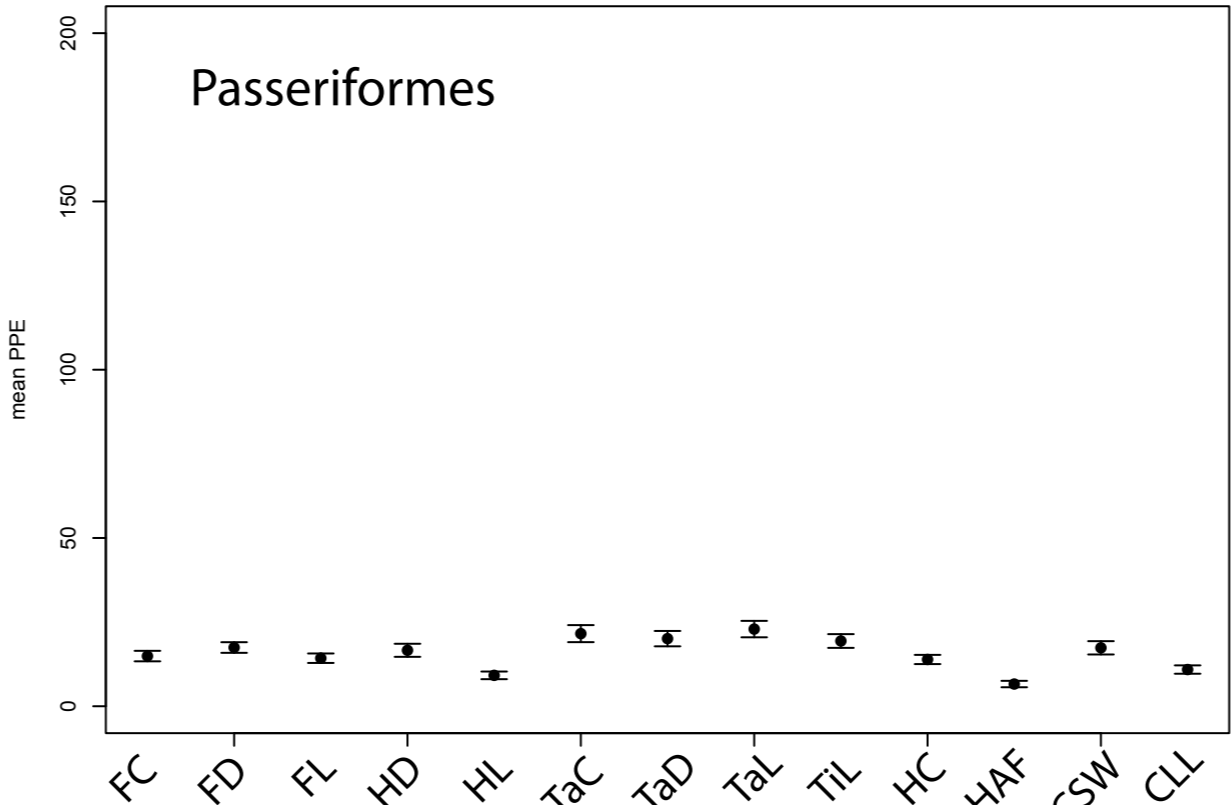

12

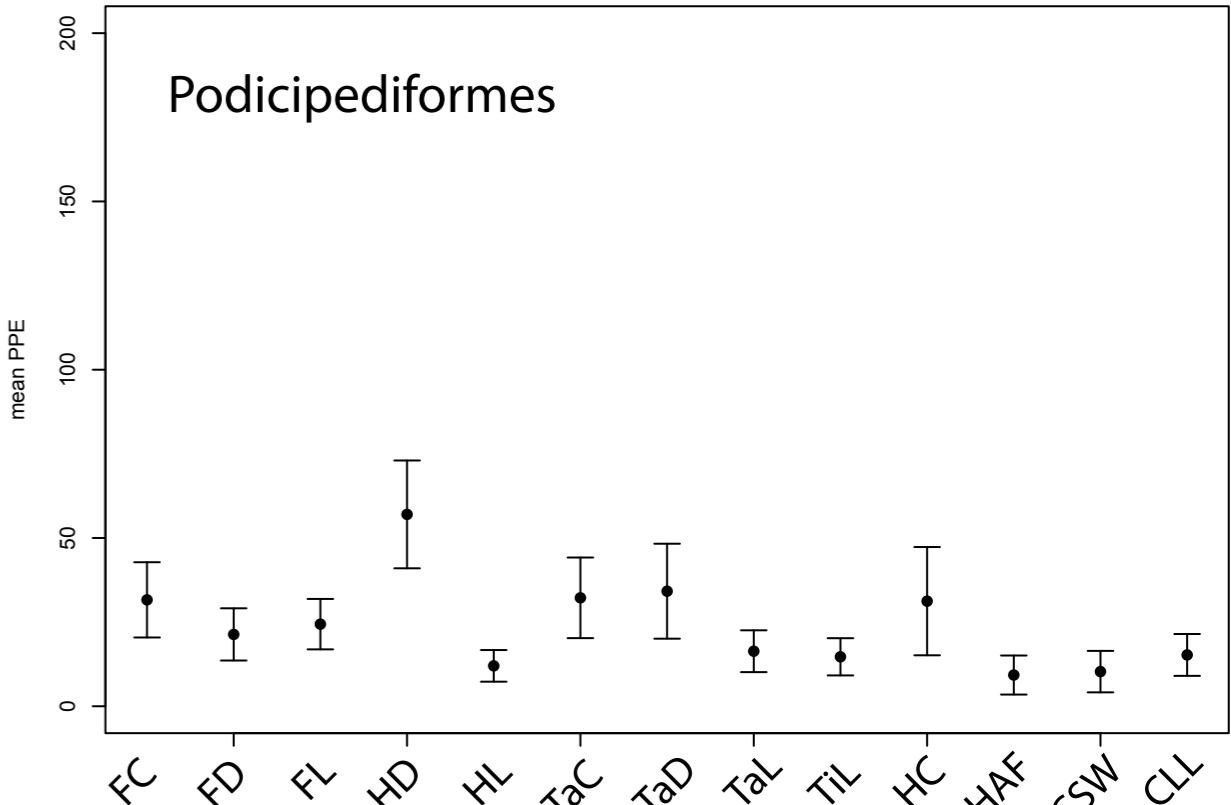

13

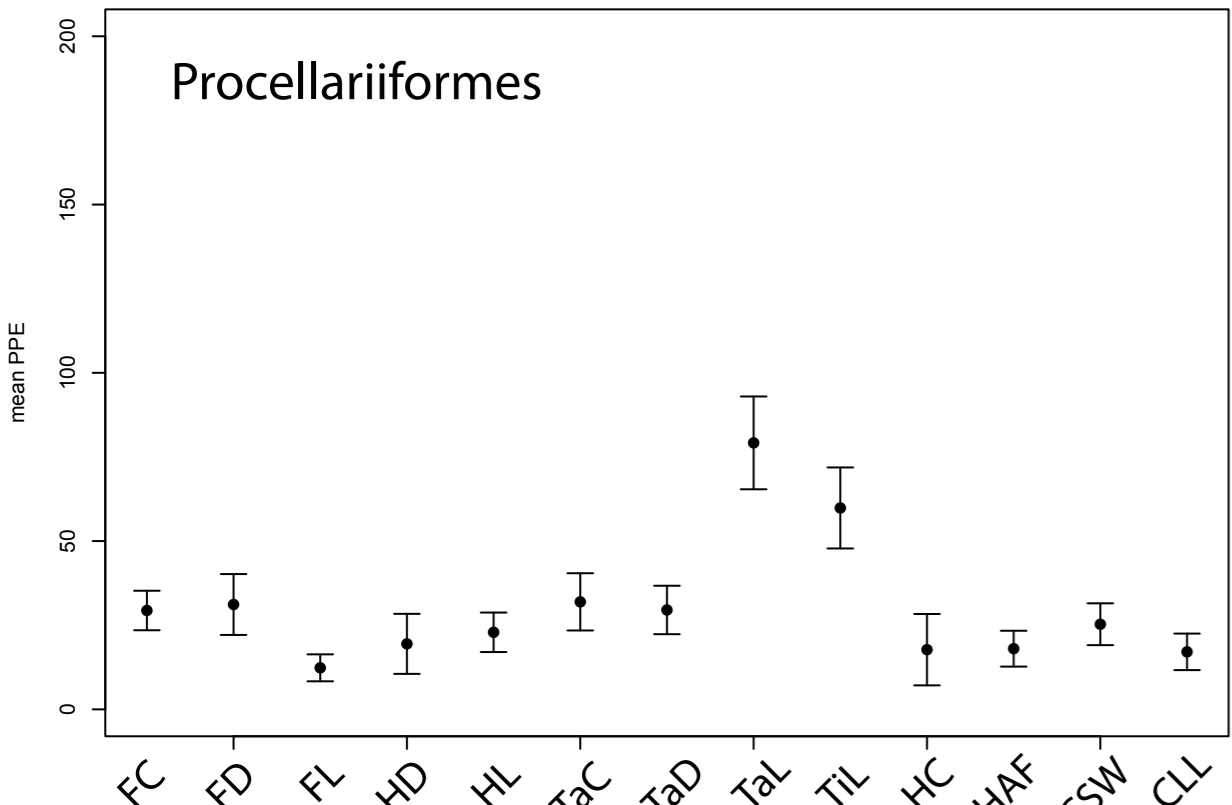

14

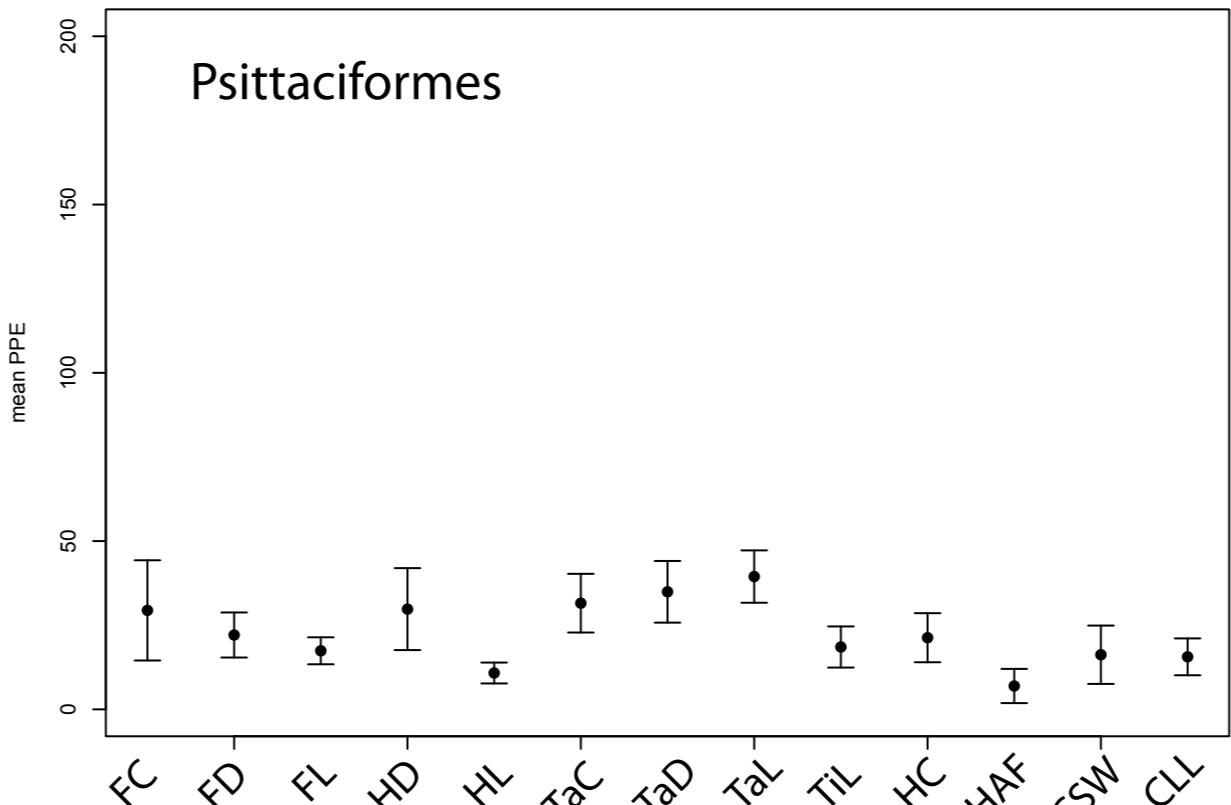

15

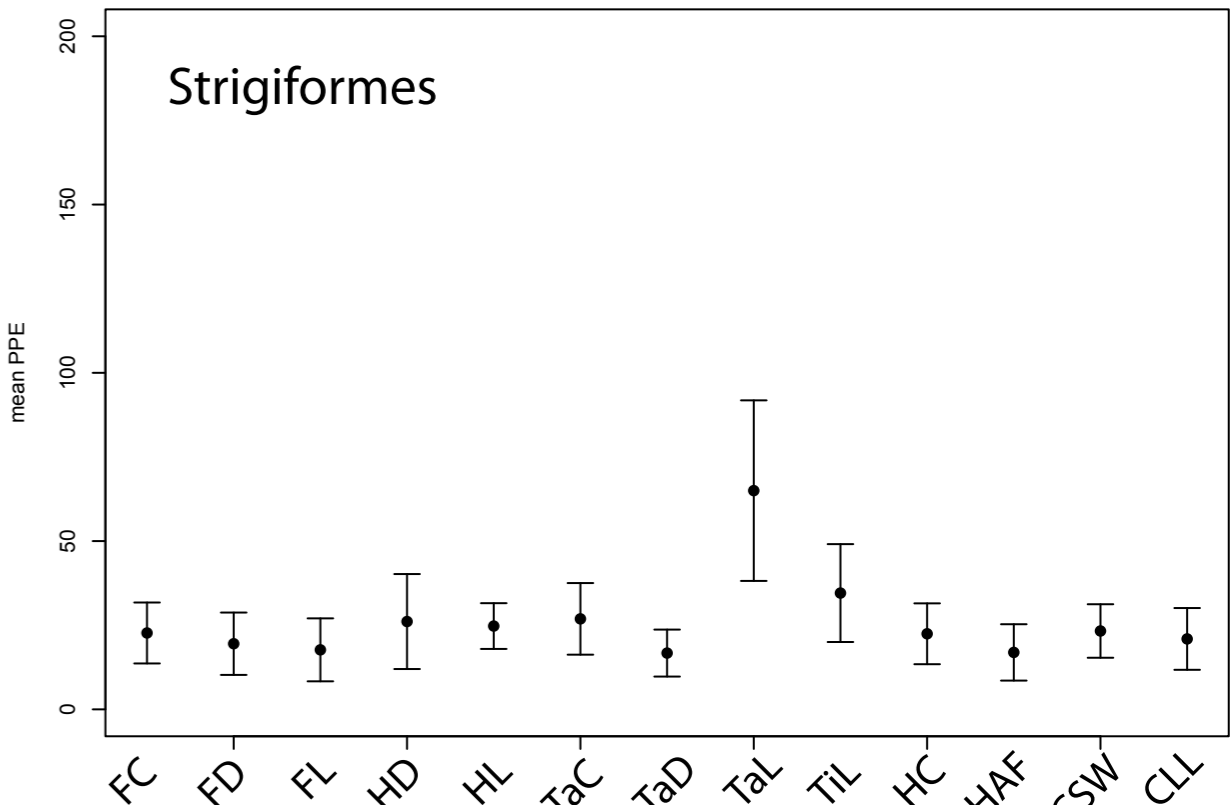

16

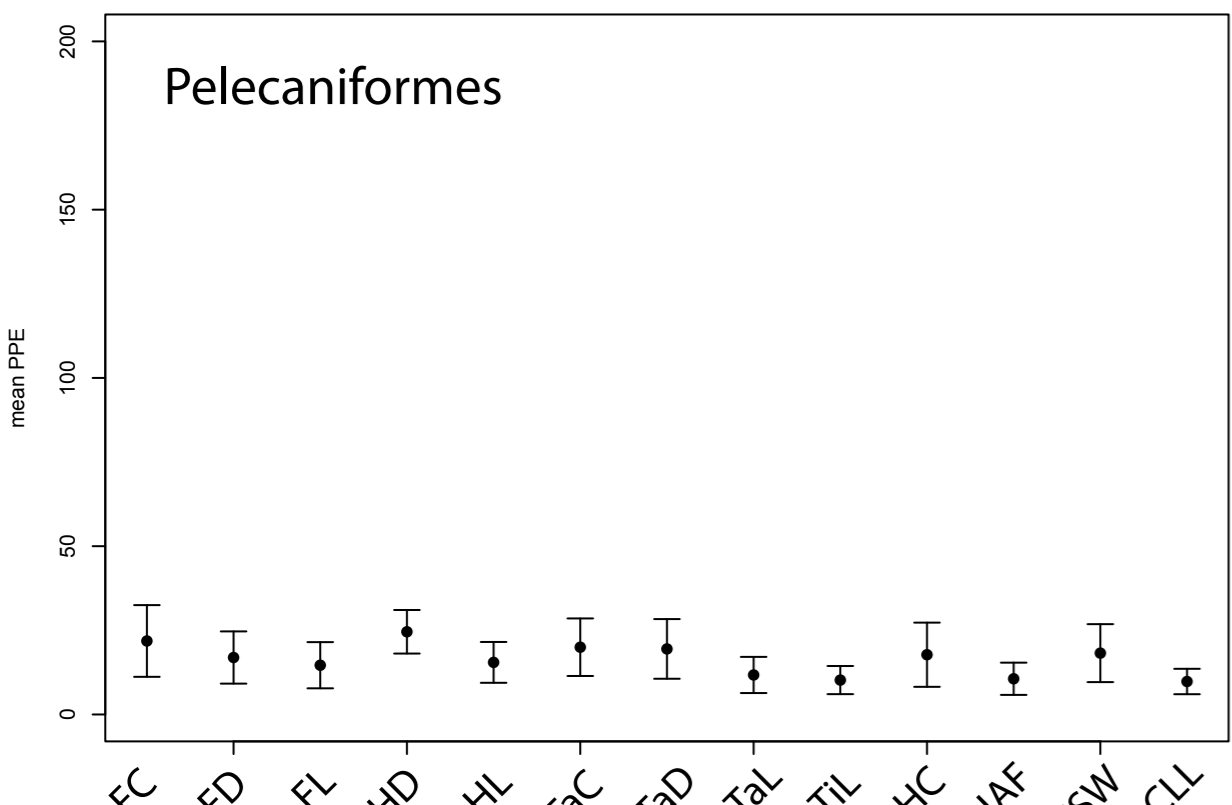

17

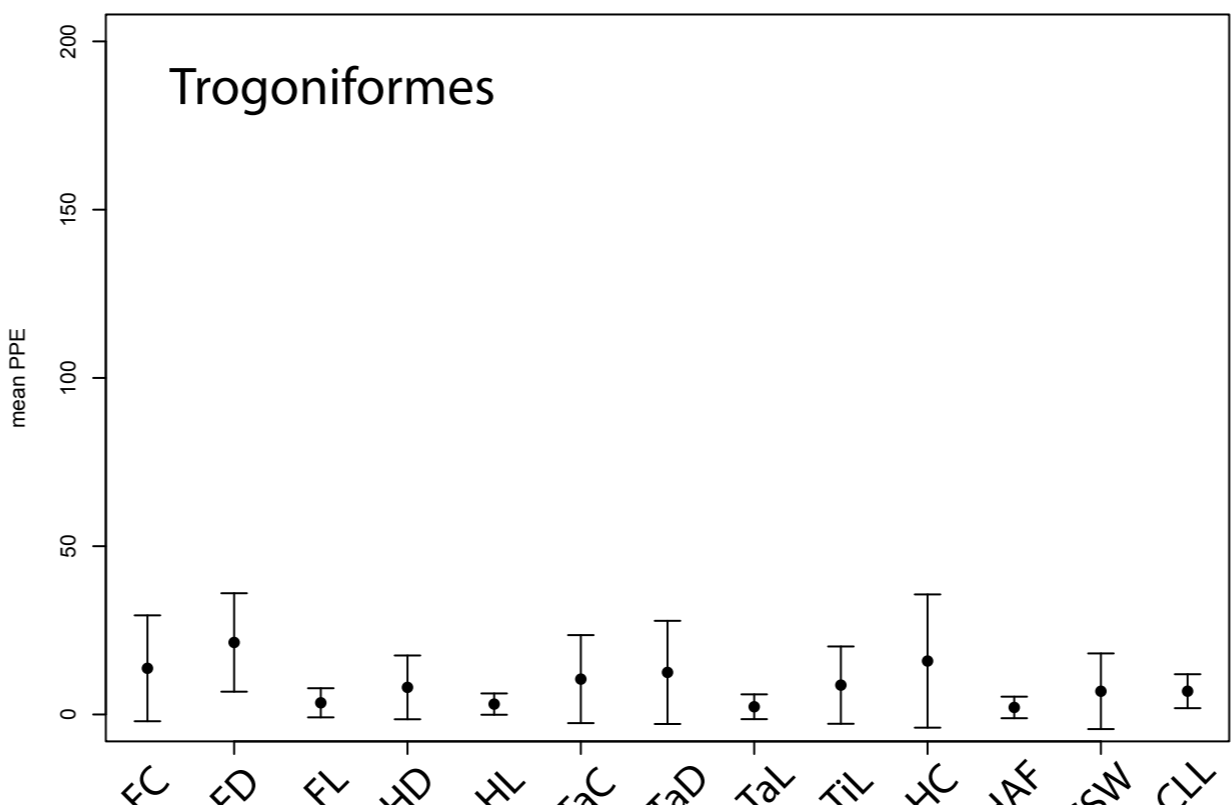

18

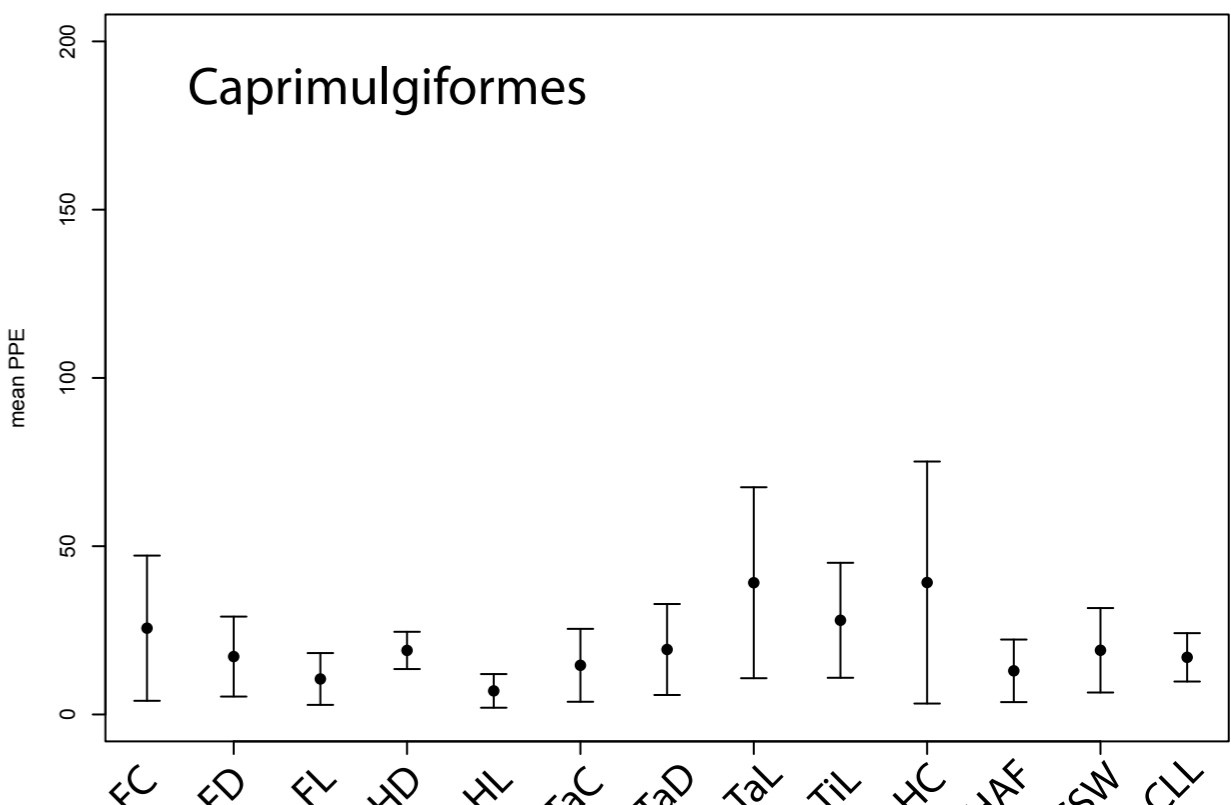

Supplement: Figure S1 — Mean PPE plots with 95% confidence intervals for 18 avian orders and 13 skeletal measurements. (PDF) [file pone.0082000.s001.pdf]
