# Supplementary material for: Skeletal Correlates for Body Mass Estimation in Modern and Fossil Flying Birds
Source: PLoS One. 2013 Nov 29;8(11):e82000. doi: 10.1371/journal.pone.0082000 (PMC3843728; doi:10.1371/journal.pone.0082000)
Supplement: Figure S2 — Mean PPE plots with 95% prediction intervals for 317 species (one specimen per species). The total dataset (317 species, 863 specimens) was pruned to 317 specimens using two random partitions. The relative ordering of body mass correlates is virtually unchanged in both cases from the total dataset. (PDF) [file pone.0082000.s002.pdf]

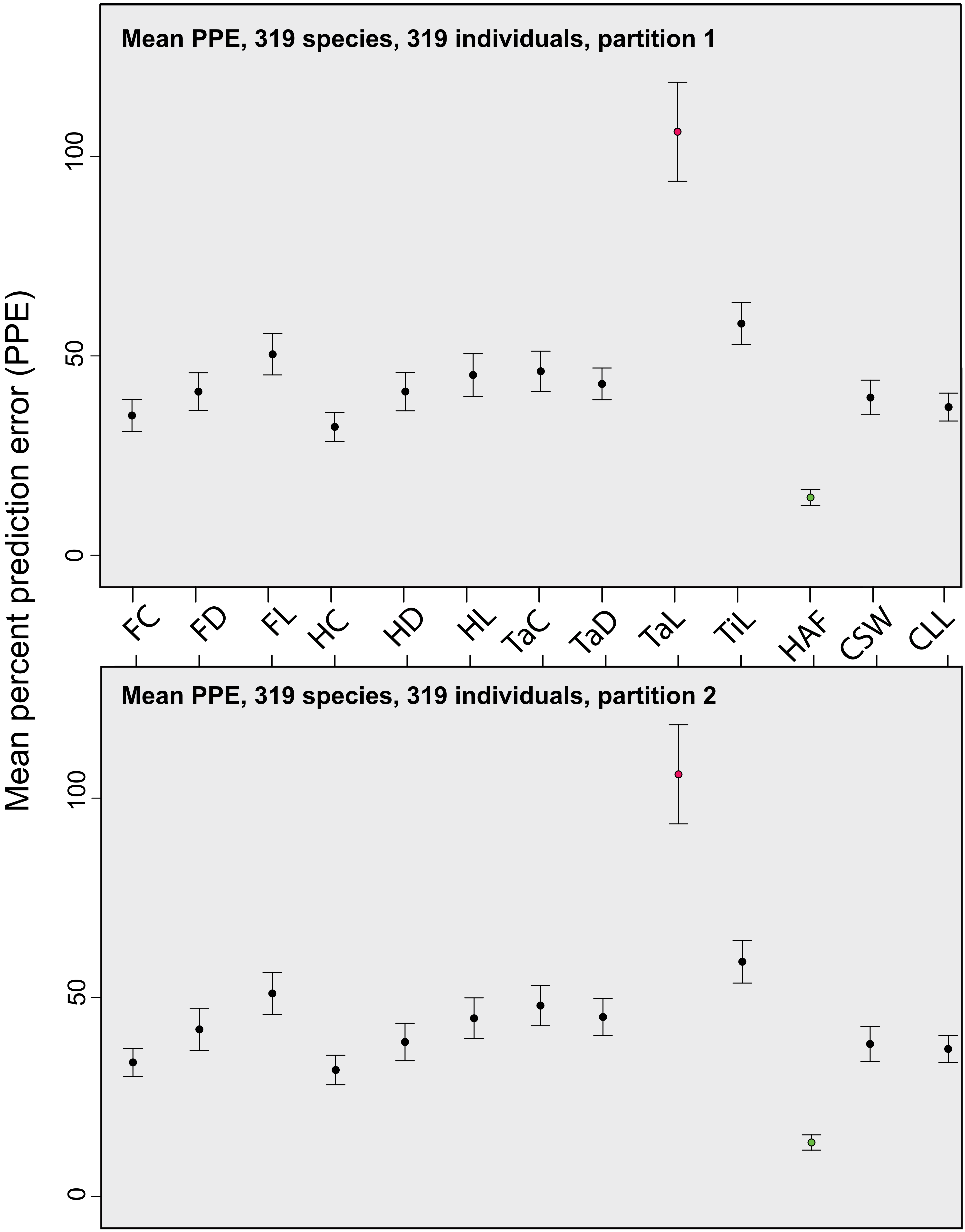

Mean PPE, 319 species, 319 individuals, partition 1
Mean PPE, 319 species, 319 individuals, partition 2
Mean percent prediction error (PPE)
0
50
100
FC
FD
FL
HC
HD
HL
TaC
TaD
TaL
TiL
HAF
CSW
CLL
